# Tissue-selective therapy of cancer

**DOI:** 10.1038/sj.bjc.6601256

**Published:** 2003-09-30

**Authors:** M V Blagosklonny

**Affiliations:** 1 Department of Medicine, New York Medical College, Valhalla, NY 10595, USA

**Keywords:** cancer, tissue, therapy, immunotherapy, hormones, resistance

## Abstract

Instead of exploiting the differences between normal and cancer cells, seemingly unrelated anticancer modalities (from immunotherapy to hormones) exploit (a) the differences between various normal tissues and (b) tissue-specific similarities of normal and cancer cells. Although these therapies are successfully used for years to treat leukaemia and cancer, their unifying principles have never been explicitly formulated: namely, they are aimed at differentiated cells and normal tissues and target both normal and cancer cells in a tissue-specific manner. Whereas tiny differences between cancer and normal cells have yet to be successfully exploited for selective anticancer therapy, numerous tissue-specific differences (e.g. differences between melanocytes, prostate, thyroid and breast cells) provide a means to attack selectively that exact tissue that produced cancer. Despite inherent limitations, such as fostering resistance and dedifferentiation, tissue-selective therapy have enormous potentials to control cancer.

As Robert Weinberg accurately pointed out, ‘virtually all the proteins made by cancer cells are normal proteins’ (Dickey, 2002). What is then a basis for immunotherapy of cancer? How immunotherapy can possibly work, if cancer cells do not provide a cancer-specific protein. What are these mysterious tumour-specific antigens? The answer is startling. For example, the melanoma-derived protein (a target of anticancer immunotherapy) turned out to be a normal (melanocyte-differentiation) antigen (Wang *et al*, 1996). So, it is not a cancer-specific protein, which is absent in normal cells, but a tissue-specific protein that is shared by both normal and malignant melanocytes. Even further, malignant melanocytes tend to lose tissue-specific proteins (dedifferentiation) rather than to acquire them. Many tumour antigens are normal tissue-differentiation antigens (Overwijk *et al*, 1999; Overwijk and Restifo, 2000). Consequently, immunisation with these ‘self’ antigens could induce autoimmunity. Vaccine strategies targeting tissue differentiation antigens may be valuable in cancers arising from nonessential cells and organs such as melanocytes, prostate, testis, breast, and ovary (Overwijk and Restifo, 2000). Thus, ‘anticancer’ immunotherapy is directed against normal differentiation antigens.

Yet, it is more difficult to control the immune response than to administer small molecular therapeutics. Can therapeutic agents be designed in a tissue-specific fashion, to attack normal differentiated cells? And is tissue-specific therapy applicable not only to ‘non-essential’ tissues such as breast and prostate but also to vital tissues such as lymphoid and thyroid. A therapy that is aimed at ‘something’ unique for a particular tissue (Figure 1

**Figure 1:**
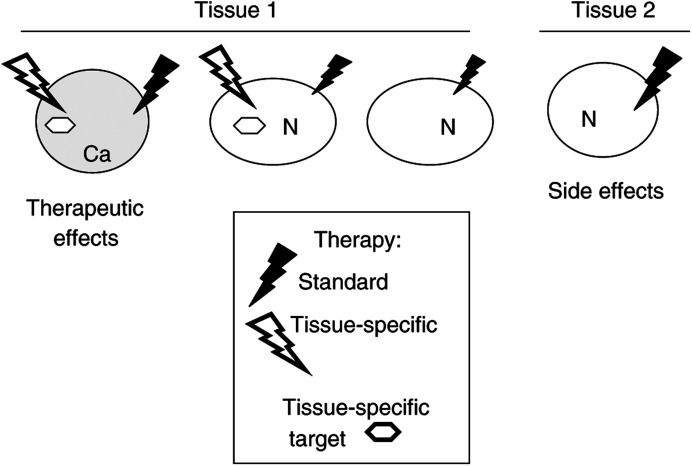
Tissue-selective and standard anticancer therapies. Standard therapy that preferentially targets proliferating cells causes side effects by targeting an essential tissue (tissue 2). Tissue-selective therapy targets cancer cells (Ca) and sub population of normal cells of the same tissue, with low side effects.

). It may be a unique cellular biochemical activity (production of melanin or uptake of iodine). It may be an antigen or an enzyme. Or cell dependence on hormones…. For example, normal prostate epithelial and testicular cells selectively depend on androgens for survival and proliferation.

## HORMONAL THERAPY IN PROSTATE CANCER

Normal prostate epithelial cells depend on androgens for their growth and survival. For example, androgen ablation induces apoptosis of prostatic glandular cells. In 1941, it was discovered that the initial growth of prostate cancer is dependent on androgens. Androgen suppression (in the form of medical or surgical castration with inhibition of adrenal androgens) became the standard primary treatment for advanced prostate cancer. Antiandrogen therapy causes regression of prostate tumours in more than 80% of patients with metastatic disease (Reese, 2000).

During the course of prostate cancer progression, however, cells convert from an androgen-dependent to an androgen-independent (Craft *et al*, 1999; Feldman and Feldman, 2001). Following androgen suppression, recurrent prostate carcinomas are able to avoid apoptosis (Koivisto *et al*, 1997). Prostate cancer cells can acquire the ability to grow without androgens by suppressing the apoptotic machinery or by using growth factors and steroids other than androgens (Feldman and Feldman, 2001). Following antiandrogen therapy, almost half patients experienced a second relapse heralded by a rise in the serum prostate-specific antigen (PSA). Tumour progression on hormonal therapy is a surrogate for impending cancer patient death (Zietman *et al*, 1996). Once a relapse occurs following primary endocrine treatment, metastatic prostate cancer is one of the most therapy-resistant human neoplasms (Zietman *et al*, 1996).

**Figure 2:**
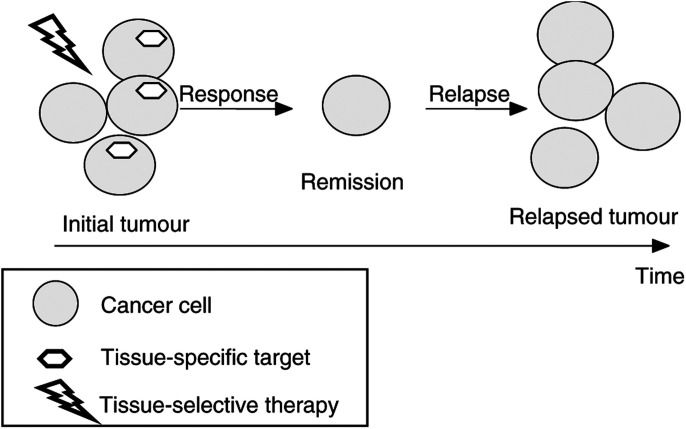
Tissue-selective therapy: from remission to relapse. By killing cells with tissue-specific targets (or traits such as hormone-dependence), tissue-selective therapy causes clinical response (remission). Cancer cells lacking tissue-specific targets survive, leading to relapse. Once relapsed, cancer is likely to be resistant to this therapy.

In summary, antihormone therapy has an excellent response (80%) but, on the other hand, promotes hormone-independent disease, which is aggressive and resistant. By killing cancer cells having a tissue-specific characteristic (e.g. hormone-dependence), tissue-selective therapy causes remission but also selects for resistant cancer (Figure 2

).

## EXPERIMENTAL PSA-DIRECTED THERAPY IN PROSTATE CANCER

Loss of one tissue-specific trait (e.g. hormone-dependence) is not necessarily associated with loss of other traits (e.g. expression of PSA, a normal prostate-specific protease). Therefore, PSA is an attractive target of tissue-specific therapy. For example, a peptide-doxorubicin ‘prodrug’, which is cleaved and activated by PSA, selectively kills prostate tumour cells *in vivo* (DeFeo-Jones *et al*, 2000).

## ANTIHORMONAL THERAPY IN BREAST CANCER

The breast is a target organ for oestrogens and progesterone, which control several functions of the normal and abnormal mammary epithelium. As demonstrated by postmenopausal hormone replacement therapy, oestrogens plus progestines induce proliferation of breast epithelial cells localised to the terminal duct-lobular unit of the breast, which is the site of development of most breast cancers (Hofseth *et al*, 1999). Treatment with antioestrogens (e.g. tamoxifen) is the first-line therapy of oestrogen receptor (ER)-positive cancer, both for metastatic disease and after mastectomy. Two-thirds of the patients with metastatic breast cancers respond to tamoxifen (or toremifen) with positive tumour regression for an average of 12–18 months. Another 20% have prolonged stable disease lasting for at least 6 months. Patients who benefited most were postmenopausal and had tumours strongly positive for both ER and progesterone receptor (Osborne *et al*, 2000). In adjuvant therapy after surgical treatment of nonmetastatic cancer, there was 50% reduction of the odds of recurrence of breast cancer.

Like other tissue-selective therapies, antioestrogens (e.g. tamoxifen) are remarkably nontoxic. Therapeutic plasma concentrations of tamoxifen are dozens times less than its maximally achievable concentrations, which is 3–8 *μ*M (Bergan *et al*, 1999). Despite the initial benefits of tamoxifen, most patients eventually relapsed (Figure 2). Furthermore, such tamoxifen-resistant tumours may be stimulated by tamoxifen and, in contrast, suppressed by oestrogens (Song *et al*, 2001). This potentially might be exploited for ‘withdrawal’ therapy in breast cancer. Similarly, in prostate cancer, intermittent androgen blockade appears to be a potential alternative to permanent androgen blockade (Wolff and Tunn, 2000).

## GLUCOCORTICOID IN LEUKAEMIA

Glucocorticoids and synthetic steroids (dexamethasone, prednisolone) suppress functions, prevent proliferation, and induce apoptosis in both T and B lymphocytes. Even during physiological stress, glucorticoids can kill CD4(+) and CD8(+) thymocytes (Ashwell *et al*, 2000). In addition to T lymphocytes, glucocorticoids induce apoptosis in B cells, pre-B and immature B cells, which is inhibited by Bcl-2 (Merino *et al*, 1994). Bcl-2 is highly expressed in B-cell precursors (pro-B cells) and mature B cells, but is low at the pre-B and immature B-cell stages of development (Merino *et al*, 1994). High levels of Bcl-2 and Bcl-x render intestinal intraepithelial lymphocytes resistant to apoptosis by glucocorticoids (Van Houten *et al*, 1997). Glucocorticoids-induced-apoptosis in cells of the lymphoid lineage at certain stages of differentiation has been exploited to a great extent in the therapy of malignant lymphoproliferative disorders. For example, glucocorticoids are included in almost all treatment regimens for childhood acute lymphoblastic leukaemia (ALL). Leukaemic blast sensitivity to glucocorticoids correlates with sensitivity to chemotherapeutic agents and with outcome after multiagent therapy (Kaspers *et al*, 1998).

Effect of glucocorticoid therapy is, however, hampered by the occurrence of resistant clones evolving under selective glucocorticoid pressure. At relapse, loss of sensitivity to glucocorticosteroids is common and out of proportion to the loss of sensitivity to other agents. Mechanisms of glucocorticoid resistance involve downregulation of glucocorticoid receptor, mutant receptor, abnormal HSPs expression, and inactivation of apoptotic pathways (Tissing *et al*, 2003).

We can generalise that tissue-selective therapy inevitably selects for resistance (Figure 2). Another lesson of glucocorticoid therapy is that tissue-selective therapy may target vitally important tissues, namely, the lymphoid system, without dose-limiting side effects. Of course, immunosuppression is an unavoidable side effect. Also, glucocorticoids cause nonlymphoid side effects, which, in theory, can be avoided by substituting glucocorticoids with antilymphocyte antibodies. Antibodies can deliver toxins and radioactivity (Allen, 2002; Frankel, 2002).

We suggest that, in essential tissues (e.g. the immune system), side effects could be further decreased by targeting cell subpopulation that originated a malignant clone (a subtissue-selective therapy). Immunotherapy of leukaemia provides examples.

## TISSUE-SELECTIVE IMMUNOTHERAPY IN LEUKAEMIA

Antibody-based therapy is intended to target leukaemia cells selectively. In reality, it targets tissue-specific antigens. Several dozens of surface antigens, referred to as CDs, are expressed on normal lymphoid and myeloid cells. Two monoclonal antibodies, rituximab (anti-CD20) and Campath-1 H (anti-CD52), are widely used. Rituximab is active in follicular non-Hodgkin's lymphomas and in chronic lymphocytic leukaemia (CLL) with response rates of 50%. It suppresses B cells without affecting T cells. Anti-CD52 antibody has notable side effects, consistent with their action against both B and T cells (Countouriotis *et al*, 2002).

## TISSUE-SELECTIVE IMMUNOTHERAPY IN MELANOMA

Like other melanoma differentiation antigens, tyrosinase-related proteins, TRP-1 and TRP-2, are expressed in melanoma, melanocytes, and retina (Wang *et al*, 1996). The immune responses to TRP-1 caused melanocyte destruction with depigmentation (vitiligo). The immune response against TRP-1 (anti-TRP-1 antibodies) could destroy both normal and malignant melanocytes (Overwijk and Restifo, 2000). The transfer, to patients with metastatic melanoma, of selected tumour-reactive T cells directed against overexpressed self-derived differentiation antigens led to regression of metastatic melanoma as well as to the onset of autoimmune melanocyte destruction (Dudley *et al*, 2002). Melanomas tend to become less pigmented in the course of malignant progression. Antimelanocyte therapy will likely facilitate this progression.

## EXPLOITING MELANIN BIOSYNTHESIS IN MELANOMA

Unique tyrosine metabolic pathways in normal melanocytes can be exploited for selective killing of melanocytes, because melanogenesis is inherently toxic and uniquely expressed in melanocytic cells. Sulphur-containing substrate (tyrosine) analogues show selective cytotoxicity towards melanoma cell lines (Thomas *et al*, 1999). Nitrosoureas-containing tyrosine derivatives are evaluated as potential antimelanoma agents (Gadjeva, 2002). Effective melanocyte-toxic therapies must cause vitiligo. This side effect is a marker of therapeutic activity.

## THYROID CANCER: ANTIHORMONE THERAPY AND RADIOACTIVE IODINE

The thyrotropin (TSH) hormone regulates proliferation and thyroid-specific gene expression (differentiation) of thyroid follicle cells. Early thyroid tumour development is correlated with mutation of alternative genes, including Ras and the TSH receptor (Wynford-Thomas, 1997). Thyrotropin suppression therapy (thyroxine application) of differentiated thyroid cancer is supported by most clinical studies (Pujol *et al*, 1996). In one-third of the cases following TSH-suppression therapy (and radioiodine therapy), dedifferentiation is observed, giving rise to tumours that are refractory to conventional treatment (Figure 2). Eventually, this may lead to the most malignant human tumour (anaplastic thyroid carcinoma) with a life expectancy of only a few months.

Although p53 mutations are present almost exclusively in poorly differentiated thyroid tumours (Wynford-Thomas, 1997), p53 mutation alone is not sufficient to drive progression of thyroid cancer to the aggressive anaplastic form (Wyllie *et al*, 1999).

Thyroid cancers are classified as papillary (82%), follicular (8%), medullary (9%), and anaplastic (1%). Anaplastic thyroid cancer cells are characterised by the absence of expression of thyroid-specific genes (TSH receptor, thyroglobulin, and thyroperoxidase). This results in their inability to incorporate radiactive iodine. Uptake of radioactive iodine, a normal function of thyroid cells, allows its accumulation 2000 over serum levels, thus killing the thyroid cell selectively. The prognosis of patients with papillary, the most differentiated thyroid cancer, is favourable and depends on tumour differentiation and the ability to take up radioactive iodine. The overall cure rate of radioiodine therapy (after 12 years) is 50% (papillary 65% *vs* follicular 23%) (Pelikan *et al*, 1997). Lacking thyroid-specific functions dedifferentiated tumours are inaccessible to standard therapeutic procedures such as radioiodide therapy and thyrotropin suppression.

**Figure 3:**
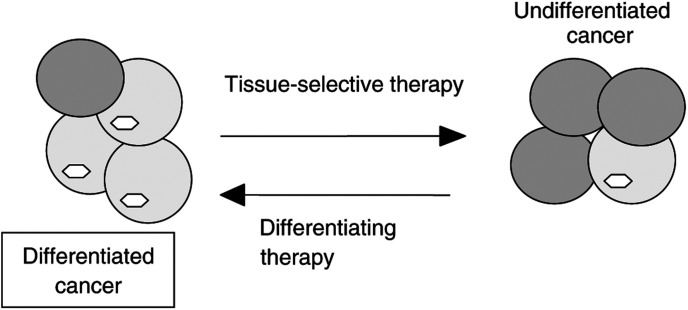
Alternating tissue-selective and differentiating therapies. Tissue-selective and differentiating therapies complement each other. Tissue-selective therapy kills differentiated cancer cells (cells having tissue-specific traits) but may promote undifferentiated cancer, which is insensitive to tissue-selective therapy. Then, differentiating therapy is needed. By reactivating tissue-specific genes, differentiating therapies may permit a new cycle of tissue-specific therapy.

We can conclude that tissue-selective therapy (anti-TSH and radioactive iodine) is very effective in differentiated cancers but can select for undifferentiated cancer cells (Figure 2). Therefore, it is feasible to alternate tissue-selective and differentiating therapies (Figure 3

).

## COMPLEMENTARY THERAPIES: DIFFERENTIATING AND TISSUE-SELECTIVE THERAPY

Retinoic acid (RA) causes partial redifferentiation and increases iodine uptake in thyroid carcinoma cell lines. In clinical studies, about 40% of the patients responded to RA with an increased radioiodide uptake (Schmutzler and Koehrle, 2000; Schmutzler, 2001). Also, histone deacetylase (HDAC) inhibitor, FR901228, can modulate the expression of thyroid-specific genes. In follicular and aplastic thyroid cancer cell lines, a low concentration of FR901228 (1 ng ml^−1^) increased both thyroglobulin, the transporter for iodine and radioiodine accumulation (Kitazono *et al*, 2001). FR901228 was suggested to be used clinically in thyroid carcinomas that are unable to trap iodine as an adjunct to radioiodine therapy (Kitazono *et al*, 2001). In an anaplastic cell line, doxorubicin, a DNA-damaging drug, induced morphologically differentiated phenotype (Blagosklonny *et al*, 1998). It has been reported a conversion of non-iodine-concentrating thyroid carcinoma metastasis into iodine-concentrating foci after chemotherapy. A patient with metastatic papillary carcinoma was treated with cisplatin and doxorubicin. Repeat ^131^I imaging after three cycles of chemotherapy showed significant ^131^I uptake in previously non-iodine-concentrating lesions (Morris *et al*, 1997). Suppression of TSH and radioiodine are highly effective in differentiated thyroid cancer. On the other hand, they eventually select for undifferentiated cancer, insensitive to tissue-selective therapy. Differentiating therapies (RA, HDAC inhibitors, doxorubicin) may induce redifferentiation. By reactivating tissue-specific genes, differentiating therapy permits a new round of tissue-specific therapy (Figure 3). Tissue-selective and differentiating therapies complement each other (Figure 3).

## PERSPECTIVES OF TISSUE-SELECTIVE THERAPY

Standard therapy of cancer is limited by its toxicity to normal cells (side effects). Logically, the goal of anticancer drug development is to exploit the differences between normal and cancer cells. Both hormonal therapy and immunotherapy were, historically, *intended* to target cancer cells. It is believed that ‘the goal of monoclonal antibody therapy is to target specific cell surface antigens on malignant hematopoietic cells, while sparing normal cells and tissues' (Countouriotis *et al*, 2002). In reality, tissue-specific therapy will preferentially spare leukaemia cells, given their tendency towards dedifferentiation. For example, a number of CD20 receptors (one of the targets of immunotherapy) is 10 times lower on leukaemic cells than on normal B cells (Rossmann *et al*, 2001).

Tissue-selective therapy exploits peculiarities of normal tissues: incorporation of iodine by thyroid tissue, dependence on androgens by prostate tissue, expression of CD20 by lymphocytes, just to name a few. Potentials in the development of antitissue therapies are enormous, because differences between tissues are diverse and profound. A breast cell can produce milk, for instance, whereas a gastric cell is capable to secrete a gastric acid. These different biochemical pathways can be, in principle, targeted pharmacologically, by prodrugs, for instance. Research in biochemistry of normal tissues, not cancer cells, is needed to facilitate development of tissue-selective therapy.

By definition, tissue-selective therapy targets normal and cancer cells of the same tissue, without affecting other tissues. Targeting nonessential tissues results in few side effects. Even side effects of tamoxifen in breast cancer patients are caused by tissue-nonselectivity of tamoxifen (e.g. effects on brain and endometrium). Furthermore, therapies against essential tissues (e.g. lymphocytes) are effective, even though they cause tissue-specific side effects (e.g. immunosuppression). To decrease side effects, novel therapy should target a subtissue instead of an entire tissue (e.g. a subpopulation of lymphocytes).

Not side effects but development of resistance, which may be accompanied by dedifferentiation, is an inherent limitation of tissue-selective therapy (Figure 2). Once relapsed, such cancer is resistant and aggressive. However, acquiring drug-resistance is a hallmark of all selective and effective therapies, including Gleevec for the therapy of CML and antibiotics for bacterial infections. This is predictable (Blagosklonny, 2002). Similarly, by killing differentiated cancer cells, which have tissue-specific traits, tissue-selective therapy ‘causes’ dedifferentiation of cancer. Therefore, tissue-selective therapy should be complemented with differentiating therapy (Figure 3) and other therapeutic approaches (e.g. antiangiogenic and standard therapy). Then, by causing remissions without severe side effects, tissue-selective therapy may control cancer and even ‘cure’ cancer, given the limited human lifespan.
